# Effects of Different Treatment Strategies and Tumor Stage on Survival of Patients with Advanced Laryngeal Carcinoma: A 15-Year Cohort Study

**DOI:** 10.1155/2018/9678097

**Published:** 2018-06-03

**Authors:** Nima Daneshi, Mohammad Fararouei, Mohammad Mohammadianpanah, Mohammad Zare-Bandamiri, Somayeh Parvin, Mostafa Dianatinasab

**Affiliations:** ^1^Behbahan Faculty of Medical Sciences, Behbahan, Iran; ^2^Shiraz HIV/AIDS Research Center, Institute of Health, Shiraz University of Medical Sciences, Shiraz, Iran; ^3^Colorectal Research Center, Faghihi Hospital, Shiraz University of Medical Sciences, Shiraz, Iran; ^4^Department of Epidemiology, Faculty of Public Health, Shiraz University of Medical Sciences, Shiraz, Iran

## Abstract

**Background:**

Laryngeal cancer is the second most common cancer in the head and neck. Since laryngeal cancer management is a complex process, there is still no standard strategy to treat this disease in order to increase the survival rate of the patients especially among those with advanced form of the disease.

**Methods:**

A cohort study was undertaken to analyze factors predicting survival of the patients in advanced stage laryngeal cancer in the Southern Iran among all patients newly diagnosed with laryngeal cancer between 2000 and 2015.

**Results:**

Data of a total number of 415 patients who have had been diagnosed with advanced laryngeal cancer during this period was used for analysis. The patients' 1-, 3-, 5-, and 10-year survival rates were 81%, 62%, 53%, and 38%, respectively. Multivariable Cox regression analyses indicated a significant relationship between patients' survival and age at diagnosis (*P* < 0.001), disease stage (*P* = 0.002), tumor grade (*P* = 0.008), positive L. node (*P* = 0.008), and type of treatment (*P* < 0.001). As expected, treatment strategy was identified as the most effective factor in survival of the patients. According to the results, patients who undergone surgical treatment experienced a longer survival than those who received other treatments.

**Conclusion:**

This study showed that the survival of patients depends on several factors, among which, treatment strategy is the most important. Combination of total laryngectomy plus chemoradiation provides superior local control and better survival compared to either radiotherapy or chemoradiation in patients with advanced laryngeal cancer.

## 1. Introduction

Laryngeal cancer (LC) is the second most common cancer of the head and neck after skin cancer [1]. The disease is accounted for approximately 30 to 40 percent of all head and neck malignancies and 1 to 2.5 percent of all malignancies in the human body [2, 3]. In terms of gender, it is the eleventh most common cancer in men during middle age [4]. LC mostly involves the glottic area [5]. The pathological type of the tumor in the majority of cases is squamous cell carcinoma and more than 40% of the patients are in advanced stage of the disease [6].

Management of advanced stage laryngeal cancer (ALC) is so complex and difficult that no ideal treatment strategy has yet been determined for it [7, 8]. Total laryngectomy followed by radiotherapy was considered the standard treatment for ALC for many years. Patients treated with laryngectomy experienced complications such as loss of voice and decreased social abilities leading to decreased quality of life in various aspects of life [9]. As a result, many treatment centers are moving from the conventional surgical treatment (total laryngectomy) to therapeutic approaches that hopefully preserve the organ's function and survival with better quality of life for the patient [10]. Although several studies on survival of patients with LC and its associated factors suggested that survival rate of LC patient's is affected by a wide range of factors such as the primary location of the tumor, the disease stage and grade, treatment type, age, and excessive alcohol consumption [11], the issue is still under sever debate.

In Iran, like other countries, the issue of best treatment strategy and patient's survival and quality of life are matters of long debates [3]. The aim of this study was to examine the effect of different LC treatment methods along with other factors on the survival of the patients with ALC in order to analyze the effects of treatment used on the survival of the patients with a retrospective cohort design in southern of Iran.

## 2. Methods

### 2.1. Study Population

This retrospective hospital based cohort study was conducted on 415 patients with ALC. The study population consisted of all patients diagnosed with LC from 2000 to 2015 whose information was available in the cancer registration center of Namazi Hospital in Shiraz, Iran. Patients were interviewed in the hospital in a private and comfort place. Patients' clinical data and medical history were obtained from patient's medical files. Namazi Hospital is a referral center not only for cancer diagnosis and treatment but also for most chronic diseases in south of the country [12]. Ethical approval was obtained from Shiraz University of Medical Sciences ethical committee (No. 1396-01-69-15171).

### 2.2. Treatment Strategies

Surgical treatment included total laryngectomy with or without neck dissection. No patients were treated with hemilaryngectomy. Patients also received concurrent chemoradiation with weekly cisplatin (40 mg/m^2^) from the first day of radiation therapy (RT) for up to 7 cycles. Using megavoltage linear accelerator (6 MV), patients received external beam RT. Radiation therapy was conducted with two-dimensional (2DRT) technique if it was before 2010 or three-dimensional conformal (3DCRT) afterward. The primary site and regional cervical lymph nodes were treated with conventional fractionation and a total dose of 60 Gy (in adjuvant setting) or 70 Gy (in definitive setting) was delivered in 30–35 fractions (5 fractions per week). Neoadjuvant or adjuvant chemotherapy was administered in an outpatient setting and patients received a median 3 cycles of chemotherapy every 3 weeks with TPF regimen (docetaxel 75 mg/m^2^ for day 1, cisplatin 75 mg/m^2^ for day 1, 5-FU 750 mg/m^2^/day with 8-h infusion on days for 1–3). Until the end of study (latest follow-up), all patients received their prescribed treatment according to the treatment strategy.

### 2.3. Data Collection and Follow-Up

The median of the patients' follow-up was 22.90 months (10.77 to 56.73). The required information was collected using a specially designed checklist from the medical files of the patients. The interview was conducted via phone call to complete an interview administered questionnaire and define the latest disease status. For patients who passed away, the date of death was defined using the patients medical records. Moreover, a phone call was made to the first-relative of patients to confirm their current health status.

The variables under study were age, gender (female, male), diagnosis date, location of tumor, N-, M-, T-stage, pathologic type, grade, treatment, node number, and positive L. node. The TNM staging system was applied to determine the stage of cancer using* AJCC* staging,* 7thedition*. Also, based on the T-stage of the patients, T3 and T4 stages of LC were considered as advanced stage and the data were used in the study. The location of the tumor was classified according to the patient's pathology report into four categories, namely, supraglottic, glottic, subglottic, and transglottic (tumor extending beyond the larynx). The pathological types of cancer were divided into two categories, squamous cell carcinoma and other types of cancer. The therapeutic approaches to the treatment of advanced stage of LC were divided into two categories, with and without surgery. Local control of the disease and the survival of the patients were the primary and the secondary endpoints of the study. Any recurrence in the larynx and adjacent structures such as thyroid, oropharynx, hypopharynx, trachea, tracheostoma, and neck soft tissue as well as cervical lymph nodes were considered as local recurrence. Date of laryngeal biopsy was considered as the time of diagnosis. Local control was calculated from the date of diagnosis to the date of local recurrence. Overall survival was calculated from the date of diagnosis to date of death due to the related disease. For patients who were still alive at the time of interview, survival time was defined as the duration from the date of diagnosis until July 20, 2016. Patients who could not be contacted and their health status was not defined were considered as censored and the duration of time between diagnosis and the time of phone call was recorded as their survival time.

Inclusion and exclusion criteria: in this study, patients with confirmed clinically or pathologically local advanced (T3-4 and/or N1–3) squamous cell carcinoma of the larynx were included. Patients were excluded if diagnosed with metastatic disease (any T, any N, M1), with early stage disease (T1-2 N0 M0), and with pathologies other than squamous cell carcinoma were excluded.

### 2.4. Statistical Analysis

Survival rates of the 1st, 2nd, 3rd, 4th, 5th, and 10th years of survival rates were calculated and used for analysis. Univariate analysis of patient's survival in different subgroups was performed using Kaplan-Meier method with log-rank test. The overall survival rate curves were plotted to present the differences in the survival of the patients according to the effective categorical variables. The multivariable survival analysis and hazard ratio (HR) were calculated using* Cox *proportional hazard model. The variables with a *P* value less than 0.25 were included in the model and their HRs and 95% confidence intervals were reported for the risk of death from LC. The significance level in this study was less than 0.05. The data was analyzed by SPSS software.

## 3. Results

As mentioned before, of 698 patients with laryngeal cancer, 283 patients with early stage cancer, metastatic disease, and nonsquamous cell cancer were excluded. As a result, data of 415 patients with ALC was analyzed, of whom 95.9% were male.  Table 1 presents characteristics of study participants and 1-, 3-, and 5-year survival among patients diagnosed with laryngeal cancer, South of Iran, 2000–2015. The mean age at diagnosis was 61.50 ± 11.16 years in men and 66.18 ± 10.47 years in women. Regarding the location of the tumor, the glottic region (208 patients, 50.1%), the supraglottic region (113 patients, 27.2%), the subglottic region (13 patients, 3.1%), and more than one region (81 patients, 19.5%) of the patients were involved. A total number of 204 patients (49.2%) were in grade 1, 148 (35.7%) were in grade 2, and 63 (15.1%) were in grade 3. Among the participants, 355 (85.5%) patients have undergone surgical treatment and received postoperative RT or CRT.

**Figure 1:**
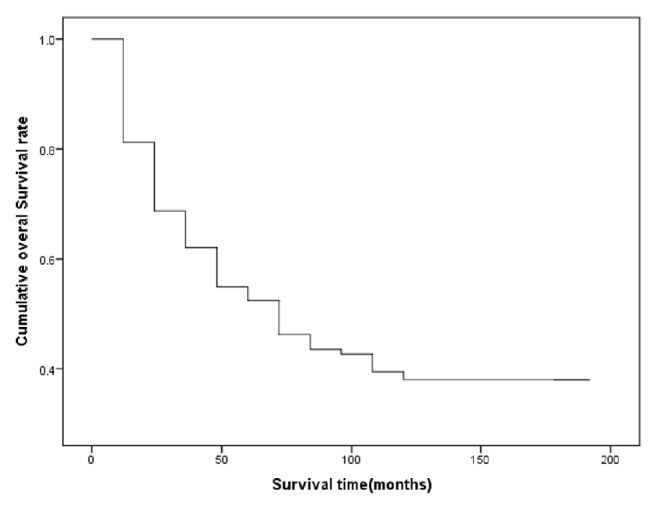
Overall survival rate among patients diagnosed with laryngeal cancer, South of Iran 2000–2015.

Figure 1 presents the overall survival rate of laryngeal cancer patients during the study period. Accordingly, the mean [±standard deviation] of survival of the patients was 38.67 [±38.07] months. In addition, the 1 to 5 and 10-year survival rates of patients with ALC were 81%, 69%, 62%, 55%, 53%, and 38%, respectively (Figure 1). The comparison of survival rates in subgroups of variables using the log-rank test showed that age (*P* < 0.001), disease grade (*P* = 0.01), stage (*P* < 0.001), node stage (*P* < 0.003), positive L. node (*P* < 0.009), T-stage (0.004), and method of treatment (*P* < 0.001) had a significant correlation with survival. That is, patients with older age at diagnosis, those with stage 4 of the disease, those with T4 of the disease, those with N1-N2 or grade 3 of tumor stage, patients who did not have surgery, and patients with positive L. node had lower survival rates. There was no significant relationship between survival rate and gender, the location of the tumor, or type of histology (*P* > 0.05).

**Figure 2:**
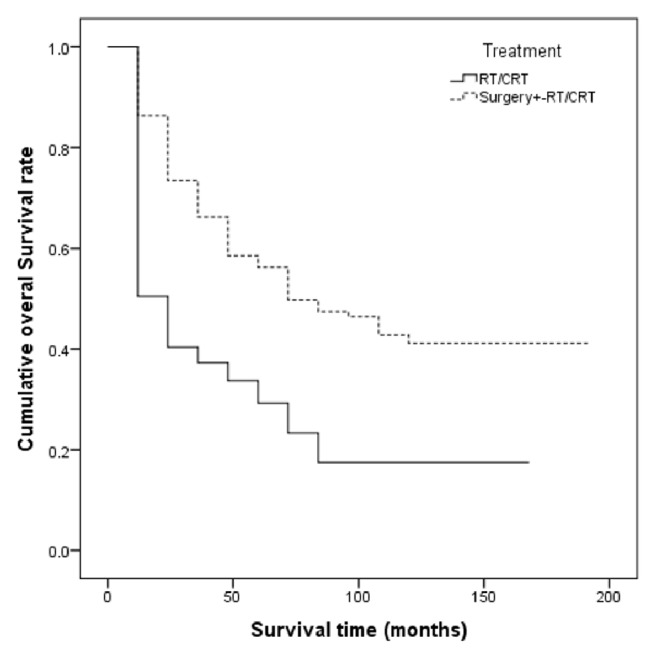
Survival rate according to treatment strategy among patients diagnosed with laryngeal cancer, South of Iran, 2000–2015 (*P* value < 0.001).

**Figure 3:**
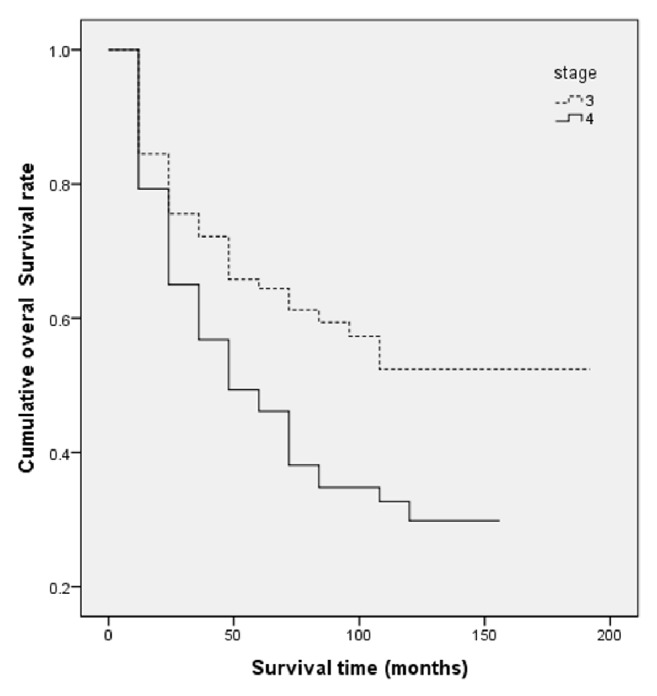
Laryngeal cancer overall survival according to different clinical stages among patients diagnosed with laryngeal cancer, South of Iran, 2000–2015 (*P* value < 0.002).

Figure 2 illustrates the survival rate of patients according to their treatment strategy. According to the results from multivariable* Cox* proportional hazard model (Table 2), age at diagnosis of cancer, type of treatment, disease stage, tumor grade, and positive L. node were effective variables in the survival of the patients. As a result, older patients were at higher risk of death. The risk of death in patients aged over 70 was 3.69 times more than those under 50 (HR_over 70 vs. less than 50 years_ = 3.76, 95% CI: 2.23–6.34, *P* ≤ 0.001). Moreover, people who undergone surgery faced a lower risk of death compared to those who received nonsurgical treatments (HR_surgery vs.   others_ = 0.32, 95% CI: 0.22–0.47, *P* < 0.001). The risk of death in people who were in grade 3 of a tumor was higher than those with a grade 1 tumor (HR = 1.71, 95% CI: 1.14–2.54, *P* = 0.008). In addition, the risk of death in patients with stage 4 of the disease was higher than those with stage 3 (HR_stage 4 vs. stage 3_ = 1.73, 95% CI: 1.22–2.45, *P* = 0.002). In addition, people who have positive L. node had lower survival rate than those with negative L. node (HR_positive L.   node vs. negative L. node_ = 1.59, 95% CI: 1.13–2.24, *P* = 0.01). Moreover, although in univariable analysis patients with T4 experienced a lower survival than those with T3, this association was not significant in the multivariate analysis. Also, Table 3 shows the measures of disease control by clinical T-stage and different treatment strategies among patients diagnosed with laryngeal cancer. Of the patients, 139 (33.5%) received surgical treatment with chemoradiotherapy. Although survival rate for this group was better than those who had surgical treatment with radiotherapy, this difference was not statistically significant (*P* = 0.12). On the other hand, there was a significant association between the survival rate of these individuals and the survival rate of those who received radiotherapy with chemoradiotherapy (*P* = 0.001).  Table 4 shows the overall survival rate by T-classification (T3, T4) and treatment strategy. Also, Figure 3 presents the graphic view of the LC cumulative survival according to different clinical stages.

## 4. Discussion

The results of this retrospective cohort study suggested that the 1st, 3rd, 5th, and 10th year survival rates in patients with LC were 81%, 62%, 53%, and 38%, respectively. The mean survival rate was 38.67 months. The results of multivariable Cox regression analysis suggested that age at diagnosis, stage of cancer, type of treatment, L. node, and tumor grade affect survival of LC patients. The log-rank test found no significant correlation between survival and gender, location of tumor, and type of histology. Rosenthal et al. calculated 5-year and 10-year survival rates as 52% and 29%, respectively [13]. Pezier et al. reported a 35.6% survival rate among American patients after 5 years of diagnosis [14]. In another study, the 3-year survival rate for advanced stage was 61% [1]. Yu et al. calculated 1-year and 10-year survival rates as 45.5% and 10.6% among Chines patients, respectively [11]. One reason for the difference in survival rate of LC patients reported in the study by Yu et al. and the present study is that patients in former study did not receive any treatments (laryngectomy, radiotherapy, and chemotherapy). Another study in the USA reported the 5-year survival rate of the patients as 53% [15].

As mentioned, comparing survival rates in different studies is not easily possible because of different methods of recruitment, design, and treatment of the patients in different studies. Univariate analysis suggested no significant statistical difference between the survival rates based on the involved region (*P* > 0.52). Some studies supported the results of this study as they also found no significant association between involved region and survival of the patients [14, 16]. In a study by Ganly et al., the involved region was significantly associated with survival of the patients only in univariate analysis and no significant relationship was observed in the multivariate model [15]. However, another study reported different results suggesting no significant difference between males and females in the survival rate of the patients (*P* > 0.53) [17]. Due to differences in the type of study, sample size, and type of LC cancer, the effect of gender on survival of patients in different studies is different. In several studies, the difference between two genders and survival of the patients was significant [17], while in others, including the present study, it was not [14, 15, 18]. In this study, node stage was identified as an important factor affecting the patient's survival.

Although the effect of node stage was significant in the univariate analysis, it was not remained in the multivariate analysis since it had collinearity with the stage of the disease in this study. A study by Gourin et al. on patients with stage 4 of LC suggested that those with a higher N stage were at a greater risk of death. Accordingly, the risk of death in people at N2 and N3 stages was 2.29 and 2.96 times more than those at a N0 stage [19]. Other studies have also supported the presence of such relationship [13–15, 18], though the relationship was not statistically significant in some other studies [1]. In multivariable analysis stage of the disease was significantly associated with survival rate, for example, the 1-, 3-, and 5-year survival rates of patients at stage 3 were 85%, 72%, and 64% and these for patients who were diagnosed at stage 4 were 79%, 57%, and 46%, respectively. Karlsson et al. reported that stage of LC is significantly associated with survival of the patients. Accordingly, 3-year survival rate in LC patients at stages 3 and 4 was 58% and 42%, respectively. The study reported the 5-year survival rate for patients at stages 3 and 4 as 47% and 32%, respectively [18]. The effect of stage of LC on survival of the patients was significant in a study by Gourin et al. The authors studied both patients with early stage in addition to patients in advanced stage of the disease. Accordingly, the 5-year survival rate for stage 3 and stage 4 of LC was 51% and 35%, respectively [19]. In another study, the 5-year survival rates of stage 3 and stage 4 were 52% and 48%, respectively. However, this relationship was not statistically significant [20].

The 1, 3, and 5 years' survival rates for T3 stage of disease were 84%, 71%, and 63%, respectively. The corresponding survival rates for patients with T4 stage were 80%, 57.5%, and 47%, respectively. Although patients with stage T4 had less survival rate, the patient's survival was not significantly affected by stage in the multivariate analysis. In a study by Pezier et al., a 5-year survival rate for T3 and T4 was 43.3% and 33.8%, respectively, which was not statistically significant [14]. Also, Timmermans et al. suggested that there was not significant difference between 5 years' survival of T3 and T4 [20].

Multivariable analysis suggested that age at diagnosis affects survival rate of the patients. In addition, the risk of death in patients aged between 50 and 70 and patients over 70 was, respectively, 1.77 and 3.69 times more than those under 50. In most studies on the survival of patients with LC, age was considered as an important risk factor. It seems that the classification of age was different in different studies which led to different results. Most studies found a significant relationship between age and the risk of death [13, 15–17], while some others found no significant relationship [14, 18].

The present study found that people with higher grades are more at risk of death, so that the risk of death in people at grade 3 was estimated to be 59% more than those at grade 1. Several studies reported different associations of which results of some were consistent with those of the present study [21] and some are contradicting [14]. Findings of the present study are in accordance with others which suggested that the selection of treatment affects the quality of life of LC patients [22].

Although total laryngectomy is a more effective treatment for LC, it significantly reduces the patient's ability to communicate and their quality of life. Recently, the combination of radiotherapy and chemotherapy has been selected as an alternative treatment strategy to help patients to retain their ability to speak [23]. Until 1990 and since after, many countries considered laryngectomy (either total or partial) as a favorable treatment for LC. However, according to a new adopted approach in order to keep the larynx organ, nonsurgical treatment is preferred [15].

The present study suggested that treatment has a significant effect on the patients' survival, as the risk of death in people who received surgical treatment was lower than those who did not. The 1-, 3-, and 5-year survival rates of patients with surgical treatment were 86%, 66%, and 56%, respectively, and the rates for nonsurgical treatment were 50%, 37%, and 29%, respectively. In general, although many studies were conducted on the impact of the type of treatment on patients' survival, they did not reach a conclusively similar result because of the differences in many aspects of their design and in the selection of the study participants. In a study that determined the effect of treatment on patients survival controlled for the effects of different factors including gender and stage, patients who received surgical treatment had significantly better survival compared to patients who did not [17]. Chen and Halpern examined the effect of type of treatment on survival of LC patients on the basis of disease stage. Patients in both stages 3 and 4 who had surgical treatment had better survival than patients with other types of treatment [9]. In one study, the investigators showed that the 5-year survival rate of LC is falling since the past decade and although the factors causing this decline are not well defined, evidence suggests that the decline in surgical treatment and a parallel increase in the nonsurgical treatments such as radiotherapy and chemotherapy have occurred during the same period of time [24]. Hoffman et al. showed that from 1985 to 1996, as an increase in the treatment of patients with nonsurgical methods (radiotherapy and CRT) was taking place, survival rate of patients was declined [25]. Another study supported the results suggesting that a significant relationship between the type of treatment and survival rate of patients exists [16]. However, several studies did not support such results [15, 18, 20].

Despite the rise in the incidence of several types of cancer (i.e., laryngeal cancer, breast cancer, and colorectal cancer) in Iran, cancer diagnosis of patients in Iranian population is commonly taking place at late stage, which adversely affects the survival of cancer patients [26–28]. In that regard, time consuming, incomplete, and selective cancer diagnosis and cancer registry in Iran are a matter of concern [29, 30]. Finally, different therapeutic approaches that apply for laryngeal cancer affect patient survival. Generally, choosing the best treatment is a complex and important process and when all factors are considered such as stage, grade, location of disease, age, and comorbidities at diagnosis, it is possible to select the best treatment and this may help to improve survival [3, 31].

*Strengths and Limitations. *To best of our knowledge, this is the first study of this type conducted in Iran. The present study used data from all LC patients during a long period of follow-up in a referral medical center in Iran. Recruiting participants who visited the biggest diagnosis and treatment center makes the results generalizable to the population of the country. However, some limitation should be taken into consideration. In this study, 355 patients underwent surgery + RT/CRT and only 60 had CRT alone. This may present some inaccuracies for a logical comparison of treatment strategies. There was no available data on diagnosis delay. Diagnosis delay can negatively affect the stage of disease [29] and, as a result, the survival of the patients [27].

## 5. Conclusion

Present study showed that various factors can affect the survival of LC patients. However, most of the associated factors are not modifiable. Early diagnosis and complete cancer registry are fundamental issues (at the national level) in better prognosis and survival of cancer patients. The more easily modifiable factor affecting the survival of LC patients is the type of treatment strategy. As mentioned above, there are several policies and approaches towards the selection of treatment strategies. Defining the optimal and standard treatment is not possible without considering the advantages and disadvantages of each strategy. Therefore, further studies on decision making over treatment selection are needed. There is always a tradeoff between a good survival rate and quality of life of LC patients in advanced stage.

## Figures and Tables

**Table 1:** Characteristics of study participants (*n* = 415) and 1-, 3-, and 5-year survival among patients diagnosed with laryngeal cancer, South of Iran, 2000–2015.

Characteristic	Category	Alive *n* (%)	Dead *n* (%)	Total *n* (%)	1-Year OS (%)	3-Year OS (%)	5-Year OS (%)
Age (year)	<50	56 (74.7)	19 (25.3)	75 (18.1)	90	79	70
	50–70	156 (62.9)	92 (37.1)	248 (59.8)	82	67	56
	>70	32 (34.8)	60 (65.2)	92 (22.2)	72	38	30

Sex	Female	13 (76.5)	4 (23.5)	17 (4.1)	93	83	60
	Male	231 (58.0)	167 (42.0)	398 (95.9)	81	61	52

T Stage	T3	111 (68.9)	50 (31.1)	161 (38.8)	84	71	63
	T4	133 (52.3)	121 (47.7)	254 (61.2)	80	57	47

Treatment	RT or CRT	24 (40)	36 (60)	60 (14.5)	50	37	29
	Surgery + RT/CRT	220 (62.0)	135 (38.0)	355 (85.5)	86	66	56

Location	Supraglottic	68 (60.2)	45 (39.8)	113 (27.2)	81	70	54
	Glottic	123 (59.1)	85 (40.9)	208 (50.1)	82	62	57
	Subglottic	8 (61.5)	5 (38.5)	13 (3.1)	100	69	42
	Transglottic	45 (55.6)	36 (44.4)	81 (19.5)	76	50	42

Stage	3	109 (69.9)	47 (30.1)	156 (37.6)	85	72	64
	4	135 (52.1)	124 (47.9)	259 (62.4)	79	57	46

Grade	1	126 (61.8)	78 (38.2)	204 (49.2)	81	64	57
	2	92 (62.2)	56 (37.8)	148 (35.6)	84	68	56
	3	26 (41.3)	37 (58.7)	63 (15.2)	75	43	32

Positive LN	No	202 (62.2)	123 (37.8)	325 (78.3)	83	67	57
	Yes	42 (46.7)	48 (53.3)	90 (21.7)	74	47	39

OS, overall survival; RT, radiotherapy; CRT, chemoradiotherapy; CT, chemotherapy; LN, lymph node.

**Table 2:** Crude and adjusted associations between the study variables and laryngeal cancer mortality among patients diagnosed with laryngeal cancer, South of Iran, 2000–2015.

Variable	Univariate			Multivariable		
	HR	95% CI	*P*-value^c^	HR	95% CI	*P*-value^c^
Age (year)						
<50	1^a^	-	-	1^a^	-	-
50–70	2.01	1.22–3.30	0.005	1.83	1.11–3.03	0.017
>70	3.90	2.32–6.55	<0.001	3.76	2.23–6.34	<0.001
Sex						
Female	1^a^	-	-	-	-	NI^b^
Male	1.36	0.50–3.68	0.54	-	-	-
Treatment						
RT/CRT	1^a^	-	-	1^a^	-	-
Surgery + RT/CRT	0.35	0.24–0.52	<0.001	0.32	0.22–0.47	<0.001
Location						
Supraglottic	1^a^	-	-	-	-	NI^b^
Glottic	1.009	0.70–1.44	0.56	-	-	-
Subglottic	0.85	0.34–2.15	0.74	-	-	-
Transglottic	1.31	0.84–2.04	0.22	-	-	-
Stage						
3	1^a^	-	-	1^a^	-	-
4	1.72	1.22–2.40	0.002	1.73	1.22–2.45	0.002
T Stage						
T3	1^a^	-	-	1^a^	-	-
T4	1.63	1.17–2.72	0.004	1.09	0.33–3.63	0.87
Grade						
1	1^a^	-	-	1^a^	-	-
2	0.92	0.65–1.30	0.65	0.92	0.65–1.31	0.67
3	1.77	1.20–2.63	0.004	1.71	1.14–2.54	0.008
Positive LN						
No	1^a^	-	-	1^a^	-	-
Yes	1.56	1.12–2.18	0.009	1.59	1.13–2.24	0.008

HR, hazard ratio; CI, confidence interval. ^a^Reference category; ^b^NI = not included (remained) in the final model. ^c^The reported *P* value is for the association of the factor with overall survival; OS, overall survival; RT, radiotherapy; CRT, chemoradiotherapy; CT, chemotherapy; and LN, lymph node.

**Table 3:** Measures of disease control by clinical T-stage and treatment groups among patients diagnosed with laryngeal cancer, South of Iran, 2000–2015.

Clinical T Stage	Treatment Group	Larynx Preservation (%)
T3	TL-R/CT	0
	RT	32
	CRT	51

T4	TL-R/CT	0
	RT	8
	CRT	29

TL-R/CT = total laryngectomy with radiotherapy with or without chemotherapy; CRT = chemotherapy; RT = radiotherapy.

**Table 4:** Overall survival rate of LC by T-classification (T3, T4) and treatment among patients diagnosed with laryngeal cancer, South of Iran, 2000–2015.

Clinical T Stage	Type of Treatment	1-Year OS (%)	3-Year OS (%)	5-Year OS (%)
T3	RT/CRT	59	44	38
	Surgery + RT	90	77	67
	Surgery + CRT	91	80	73

T4	RT/CRT	39	27	18
	Surgery + RT	83	58	47
	Surgery + CRT	85	64	54

OS, overall survival; RT, radiotherapy; CRT, chemoradiotherapy; CT, chemotherapy.

## Data Availability

The data used to support the findings of this study are available from the corresponding author upon request.

## Abbreviations

LC: Laryngeal cancer
ALC: Advanced stage laryngeal cancer
BMI: Body mass index
SD: Standard deviation
OR: Odds ratio
95% CI: 95% confidence interval
RT: Radiotherapy
CRT: Chemoradiotherapy
T: Tumor
N: Node
M: Metastasis.
